# Effect of bluetongue serotype 3 vaccines on probability of viremia and NSAID usage in Dutch dairy cattle herds

**DOI:** 10.3389/fvets.2025.1619614

**Published:** 2025-07-30

**Authors:** Reinard R. Everts, Margit Groenevelt, Kees-Jan Oosterhuis, Elske Kelderman, Gerrit Koop

**Affiliations:** ^1^Diergeneeskundig Centrum Zuid-Oost Drenthe, Coevorden, Netherlands; ^2^Dutch Sheep and Goat Breeders Association (NSFO), Zaltbommel, Netherlands; ^3^Sustainable Ruminant Health, Department of Population Health Sciences, Faculty of Veterinary Medicine, Utrecht University, Utrecht, Netherlands; ^4^Vereniging Kernpraktijken Rundvee, Harmelen, Netherlands; ^5^Dierenkliniek Stad en Westerkwartier, Zuidhorn, Netherlands; ^6^Dierenartsenpraktijk Dokkum, Dokkum, Netherlands

**Keywords:** bluetongue (BT) disease, BTV3, vaccination, viremia, NSAID

## Abstract

**Introduction:**

After the outbreak of bluetongue serotype 3 (BTV3) in The Netherlands in September 2023, three pharmaceutical companies developed inactivated vaccines against this newly emerged serotype in a short period of time, making it possible to start a vaccination campaign just before the start of the new outbreak in 2024. This study describes effectiveness of these vaccines under field conditions in Dutch dairy cattle herds.

**Materials and methods:**

Data from 1,114 dairy cattle herds was collected, of which 518 (46.5%) completed the double vaccination scheme and 29 (2,6%) vaccinated once. Results from PCR blood samples that were taken as part of the early warning system were used as a proxy for viremia. As a proxy for morbidity in dairy cattle herds, NSAID usage was used.

**Results:**

Herds that fully vaccinated with Bultavo, were less frequently tested for BTV3 and we observed a significant reduction in the probability of detecting bluetongue virus RNA compared to non-vaccinated herds. A significant increase in NSAID usage in the months July, August, September compared to the same period the year before was seen in non-vaccinated herds. Such increase was also seen in vaccinating herds, but a significantly smaller increase was seen for Bultavo vaccinating herds.

**Discussion:**

Altogether, we found large differences between the three vaccines in field effectiveness in dairy cattle in 2024 and conclude that Bultavo is the preferable option to prevent clinical signs and disease transmission under these circumstances.

## Introduction

In the beginning of September 2023 The Netherlands faced a rapidly spreading and highly virulent outbreak of bluetongue virus (BTV) serotype 3 (BTV3) in cattle, sheep and goats (1–3). The outbreak resulted in 4,463 PCR positive herds by the end of 2023, mainly in the center of the country around the index case (4); 50% of these positive herds were cattle herds, 48% sheep flocks and 2% were goat or alpaca herds (3). Pharmaceutical companies soon started developing inactivated vaccines against this newly emerged serotype. By May 2024, three inactivated BTV3 vaccines were approved under exceptional circumstances ex art. 110 EU (2) by the Dutch government. Other European countries soon followed. In the procedure for approval, the manufacturers showed safety and first proof of efficacy of the vaccines based on experimental infections in controlled environments with small groups of animals. These vaccines came available on the Dutch market early May (Syvazul, Syva, Spain), end of May (Bultavo, Boehringer-Ingelheim, Germany) and beginning of June (Bluevac, CZ Laboratories, Spain). Uptake of BTV3 vaccination in 2024 in Dutch dairy cattle herds was estimated at 45% of the herds, but only 72% of the herds that vaccinated had their herd vaccinated twice as per data sheet (5).

In 2024, the first officially reported case of BTV3 in The Netherlands was reported on June 14^th^ (6) and more cases followed quickly, leading to a total of 8,937 PCR positive herds in 2024 (7), with a distribution over cattle, sheep, goat and alpaca herds of, respectively, 54, 40, 5 and 1% (6).

From an epidemiological point of view, an effective vaccine should prevent viremia and thus minimize the risk of transmission of the virus from cow to vector (horizontally) or from cow to fetus (vertically). For the strain of this outbreak (BTV3/NET2023), it was shown that dairy cattle can remain PCR positive up to 28 weeks after infection, with low Ct values for at least 15 weeks after the start of the monitoring (8). Vertical transmission of BTV3 was not seen in every infected herd (8), but in some infected herds it was observed (2) and this has also been described for BTV8 (9, 10). Without total or at least substantial reduction of viremia, there is a great risk that the BTV3/NET2023 strain of BTV will become endemic in large parts of Europe, since every year new naïve susceptible hosts are born.

Furthermore, reduction of clinical signs is considered important to assess the value of BTV vaccination (11).

It is, however, unknown to what extent the available vaccines prevented viremia or clinical signs under field conditions, and whether the available vaccines were equally effective. Therefore, we collected data from 1,114 dairy cattle herds served by eight veterinary practices in The Netherlands, located in regions where the BTV3 prevalence was low in the beginning of 2024. The aim of our study was to quantify the effect of the three available vaccines on the probability of viremia and on herd-level non-steroidal anti-inflammatory drugs (NSAID) usage as a proxy for clinical disease.

## Materials and methods

### Data collection

Eight veterinary practices, affiliated with the association ‘Kernpraktijken Rundvee’ (Harmelen, The Netherlands), were enrolled in this study. All data used was owned by the veterinary practices and was anonymized before analysis. The eight practices were located in regions where the within-herd animal prevalence against BTV3, based on bulk milk antibodies, was <20% by the beginning of 2024 (12). These regions were chosen to minimize the possibility of interference with natural immunity due to exposure in 2023 as much as possible.

Data from the dairy cattle herds that were serviced by these veterinary practices were collected through their practice management systems for sales data on NSAIDs and vaccines and first two digits of the postal code area. As Dutch farmers are required to enter into an agreement with a veterinary practitioner and have no other means for purchasing such medication, both purchase of NSAIDs and vaccines at the practice of this practitioner is therefore a reliable reflection of the total amount of these drugs that were used in a certain period and since vaccinations in cattle should be carried out by a veterinarian, the invoice date reflects the actual vaccination date. Data on animals per farm was extracted from the database of the Royal GD, Deventer, The Netherlands. Only farms with the registration ‘dairy’ in that database were used, to exclude youngstock rearers and beef herds.

### BTV testing and NSAID usage

Two proxies of vaccine efficacy were studied. First, positive PCR results for BTV3 in blood samples collected as part of the early warning system served as a proxy for viremia. The early warning system was implemented in The Netherlands shortly after the start of the outbreak and allowed veterinarians to submit up to three EDTA blood samples per herd at one point in time to the National Reference Laboratory (Wageningen Bioveterinary Research, Lelystad, The Netherlands) from animals with clinical signs indicative of BTV infections, so spread of BTV3 could be monitored. For the farmer, there were no further costs for analysis. There was no obligation to test animals if they moved off a farm or before vaccination. For this early warning system, two subsequent panBTV real-time PCR assays are used (13). In several cases, samples from the same herd were sent in at multiple points in time. A herd was considered test positive if at least one animal in the herd tested PCR positive, regardless of the number of animals tested per herd. Data on PCR results were provided by seven of the eight participating practices.

Second, usage of NSAIDs was used as a proxy for morbidity. Sales data on NSAID purchase per herd for the months July, August and September (q3) of both 2023 and 2024 from all eight practices was used. NSAID are the only family of analgesics available for dairy cattle in the Netherlands and are classified as ‘prescription by a veterinarian only’. Products that combined NSAID with other drugs (e.g., NSAID and antibiotics) or NSAID that had no registration for milk producing cattle were excluded. Also, we did not include other anti-inflammatory drugs (e.g., corticosteroids) in our dataset. For NSAID with the active component ketoprofen and flunixine, a duration of action of 24 h was assumed. For products with the active component meloxicam and carprofen, a duration of action of 72 h was used. We assumed an average weight per dairy cow of 675 kg for calculation of the average number of daily dosages, corresponding to a Holstein Friesian in the second lactation (14), the predominant breed in The Netherlands (15, 16). All purchased NSAIDs were allocated to the number of dairy cows present, so any use possible in youngstock present was attributed to use in adult dairy cows. For the number of animals per herd, the total number of dairy cows in June 2023 and June 2024 were used as reference for herd size. With this, we calculated the average number of treatment days per 10 cows in q3 of 2023 and in q3 of 2024.

### Vaccine data

For the information on bluetongue vaccinations, the invoice data of all eight practices from May 1^st^ until September 30^th^ 2024 were included in the dataset. If a herd had more than two vaccination dates, we assumed that the date with the largest number of vaccinated animals concerned the vaccination of the dairy herd and those dates were used to determine the date of first and second vaccination. The number of doses sold was checked against the herd size. Where the number of used doses resulted in a percentage of <70% of the present adult cattle in June 2024, it was assumed that only youngstock was vaccinated. Such cases were assigned to the non-vaccinated group. Six out of eight practices provided data for all herds and one practice about part of the herds about grazing, which could be a potential confounding variable of the vaccination effect. This variable had 3 levels: full grazing (at least 720 h a year), partial grazing (only youngstock or dry cattle) or year round housing.

### Statistical analysis

Vaccination status per herd was recoded into a five level categorical variable: not vaccinated, Bluevac, Bultavo, Syvazul and a single dose. In two cases, the first vaccination was done with Syvazul and the second vaccination was done with Bultavo. These cases were classified as Bultavo-vaccinated.

To determine the effect of vaccination on probability of testing for BTV3 and on probability of testing positive for BTV3 (among tested herds), two mixed logistic regression models were built, with being tested (yes versus no) or test-outcome (positive versus negative) as the dependent variables and a random effect for veterinary practice. Potential confounding was assessed for the following variables: 2-digit postal code area (as categorical variable), herd size (continuous) and grazing (as defined above). Variables were deemed confounders if inclusion of that variable changed the beta-estimate of other statistically significant levels of the vaccination effect changed by >20%.

The effect of vaccination on NSAID usage was tested in a linear mixed model. The same variables as mentioned above in the logistic regression model were included to identify potential confounders and veterinary practice was used as random effect. The dependent variable in this model was the difference in the average number of treatment days per ten cows per herd between q3 of 2024 and of 2023. All analyses were performed in R version 4.4.0 (17), and mixed models were built using the lme4 library (18).

## Results

### Association between vaccination status and BTV3 testing

Approximately half of the herds in our sample were vaccinated. Bultavo was used most frequently, followed by Bluevac and then by Syvazul. In most herds (76%), the second vaccination was given 21 to 28 days after the first. In 12% the interval was less than 21 days and in 13% the interval was between 29 and 62 days. Overall, about one third of all herds tested for BTV3 and the majority of samples tested positive (Table 1). Herds vaccinated with Bluevac or Syvazul, however, had significantly higher odds of being tested than non-vaccinated herds, whereas Bultavo vaccinated herds had similar odds as non-vaccinated herds. Information on grazing was available for 725 herds in 2024 and 463 of these herds (59%) did full grazing, 56 partial grazing and 206 (28%) housed their cows permanently in 2024. Including grazing in the model for probability of testing for BTV3 increased the OR for Bluevac, but did not affect the other estimates. Surprisingly, full grazing was associated with lower odds of testing compared to no grazing.

**Table 1:** Vaccination status of 816 Dutch dairy cattle herds within 7 veterinary practices and associated proportion of herds tested for bluetongue virus serotype 3 (BTV3) and proportion of test positives among tested herds during 2024.

Vaccination status	Herds	Tested		Test positive	
		*n*	Proportion (95% CI)	*n*	Proportion (95% CI)
Not vaccinated	398	173	0.43 (0.39 to 0.48)	165	0.95 (0.91 to 0.98)
Bluevac	110	72	0.65 (0.56 to 0.74)	69	0.96 (0.88 to 0.99)
Bultavo	248	90	0.36 (0.31 to 0.42)	33	0.37 (0.27 to 0.47)
Syvazul	37	30	0.81 (0.66 to 0.91)	27	0.90 (0.74 to 0.97)
Single dose	23	15	0.65 (0.45 to 0.81)	15	1.00 (0.80 to 1.00)
Total	816	380	0.47 (0.43 to 0.50)	309	0.81 (0.77 to 0.85)

The proportion of test positive samples among tested herds was significantly lower in herds vaccinated with Bultavo, whereas Bluevac and Syvazul vaccinated herds did not differ from the non-vaccinated reference herds (Table 2). These associations were not affected by any of the tested potential confounders.

**Table 2:** Odds ratio (OR) of the association between vaccination status and dairy cattle herds being tested (based on 708 herds in 7 veterinary practices) and testing positive (defined as at least one animal in a herd being test positive by PCR, based on 365 tested dairy cattle herds in 7 veterinary practices) for Bluetongue virus serotype 3, in The Netherlands. Results from two mixed logistic regression model with a random practice effect, excluding herds that were vaccinated only once.

Variable	Level	Tested y/n (*n* = 708 herds)			Test positive y/n (*n* = 365 herds)		
		OR	95%CI (OR)		OR	95%CI (OR)	
			Lower	Upper		Lower	Upper
Vaccination status	Not vaccinated	Reference			Reference		
	Bluevac	2.97	1.43	6.23	1.05	0.25	4.48
	Bultavo	0.79	0.54	1.19	0.03	0.01	0.07
	Syvazul	5.58	2.14	14.73	0.38	0.09	1.56
Pasture access	No	Reference					
	Partial	1.51	0.78	2.92			
	Full	0.67	0.46	0.98			

### Association between vaccination status and NSAID usage

**Figure 1:**
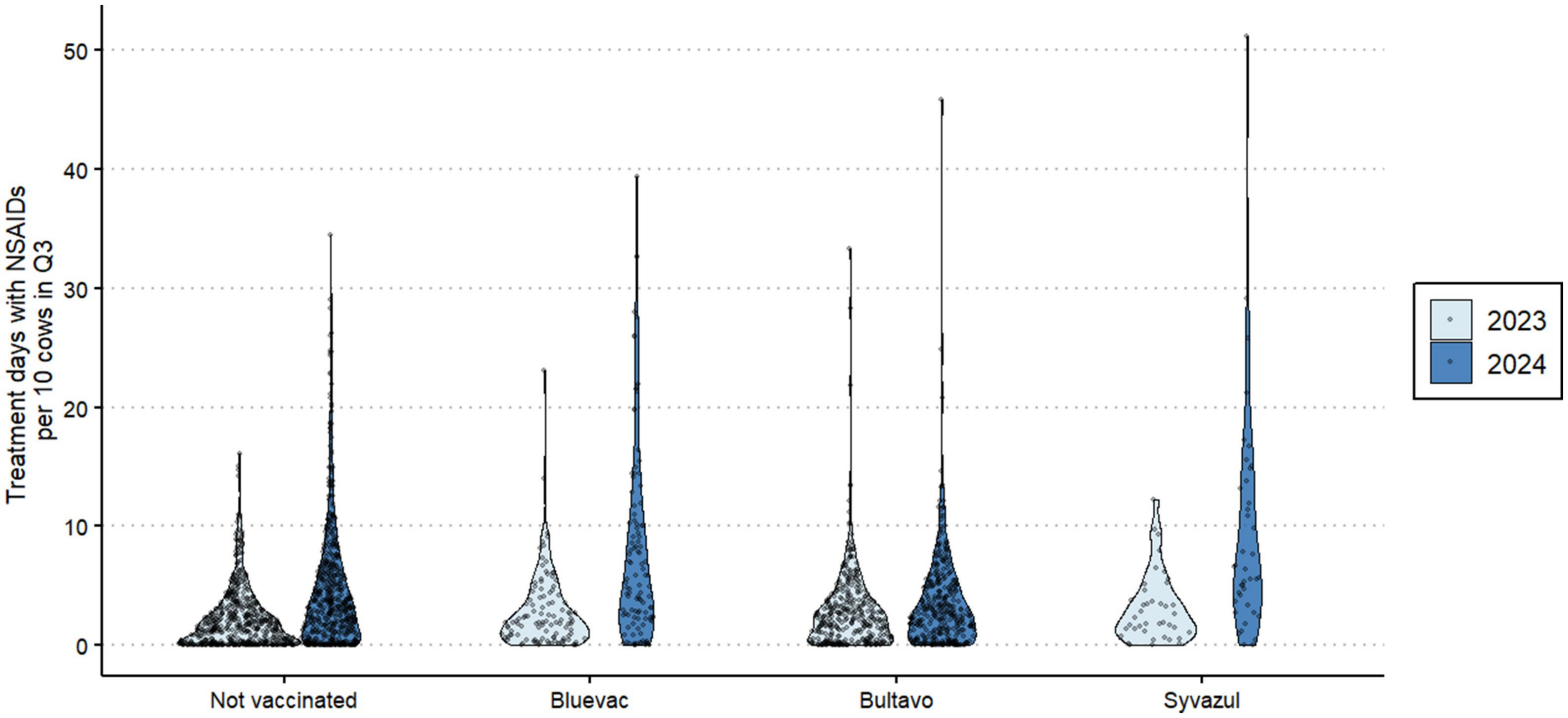
Association between vaccination status in 2024 against Bluetongue Virus serotype 3 and distribution of herd-level average NSAID usage (treatment days per ten cows) in q3 of 2023 and q3 of 2024 in 1,085 Dutch dairy cattle herds. In q3 2023, these herds had had no or limited exposure to BTV3 and herds were vaccinated against BTV3 in May–August of 2024.

In 2023, the herds in our study had had little exposure to BTV3 (12) and first cases in the studied area were not reported in q3 of 2023 (4). NSAID usage was highly variable between farms (Figure 1), and the overall usage increased in 2024 compared to 2023. Figure 1 shows in particular that the number of farms with no or little use was less in 2024 than in 2023, except for Bultavo vaccinated herds. Interestingly, farms that vaccinated in 2024 already used on average more NSAID in 2023 than non-vaccinated herds (Figure 1). Whereas NSAID usage increased in general, Table 3 shows that Bultavo-vaccinated herds had a significantly smaller increase in NSAID usage than non-vaccinated herds, whereas Bluevac and Syvazul vaccinated herds had a significantly larger increase in NSAID usage than the non-vaccinated reference category. Grazing, nor postal code area or herd size acted as a confounder of the association between vaccination and the outcome variable in this regression model.

**Table 3:** Results from a linear mixed model on the association between vaccination status against Bluetongue Virus serotype 3 (BTV3) and the difference in NSAID usage (treatment days per 10 cows) between q3 2024 and q3 2023 in 1,085 Dutch dairy cattle herds, with veterinary practice (*n* = 8) as random effect. In q3 2023, these herds had had limited exposure to BTV3 and herds were vaccinated against BTV3 in May–August of 2024.

Vaccination status	Difference in NSAID usage 2024–2023			
	Beta	95% CI (Beta)		*p*
		Lower	Upper	
Not vaccinated (intercept)	2.59	1.97	3.20	0.00
Bluevac	1.80	0.65	2.95	0.00
Bultavo	−1.63	−2.29	−0.96	0.00
Syvazul	3.87	2.33	5.41	0.00

## Discussion

In this study, we aimed to quantify the effect of BTV3 vaccines on proxies for viremia and morbidity. Effectiveness of vaccines refers to the performance of those vaccines under field circumstances, and it reflects the proportionate reduction in disease among the vaccinated group (19). Breed composition (20), climate factors (21) and vector abundance and species (22) can alter BTV disease outcome and therefore effectiveness could vary between regions in the world. For BTV serotype 8 (BTV8), it was shown that vaccine uptake strongly influenced the severity of epidemics, with the greatest reduction achieved with high levels (95%) of vaccine uptake and outbreaks significantly smaller for high (>80%) compared with low (<70%) levels of efficacy (23). Our study showed an uptake of vaccination in dairy cattle herds in the studied region with an average of 48%; comparable to the general Dutch estimate of 45% (5). This relatively low uptake can be partly explained by the fact that timely availability of vaccines before the onset of the epidemic in 2024 was impaired. Possibly, the fact that the disease had not spread through the whole country in 2023 gave hope to farmers that it would not reach them in 2024 (24).

### Prevention of viremia

The probability of being tested for BTV3 was higher in Bluevac and Syvazul vaccinated herds than in the non-vaccinated herds, whereas Bultavo vaccinated herds were not different from the reference group. The fact that Bluevac and Syvazul vaccinated herds were more likely to be tested is counterintuitive and suggests confounding. It is possible that farmers who are inclined to vaccinate are also more likely to submit samples for testing as they are more risk averse. Or farmers who are situated closer to farms that were positive in 2023 are more inclined to vaccinate and were for that reason also more likely to have clinical cases and therefore submitted more tests. The higher proportion of tested herds in Bluevac and Syvazul is therefore probably at least in part the result of a different behavior of the farmer or higher exposure to BTV3 or both and it seems implausible to be a direct result of the vaccine itself. However, Bultavo vaccinated herds can be expected to be similar to the other vaccinated herds in these aspects, suggesting that our results indicate that Bultavo gave better protection against BTV3. This is in line with the fact that among tested herds, Bultavo vaccinated herds had significantly lower odds of testing positive than the other herds. The fact that Bluevac and Syvazul vaccinated herds had no statistically significant lower odds of testing positive than the non-vaccinated herds, is insufficient as proof that these vaccines gave no protection, as the exposure to BTV3 may have differed between these herds and the non-vaccinated herds. But the fact that Bultavo did differ from non-vaccinated herds suggests that Bultavo performed better in this aspect than the other vaccines. We saw some confounding of the association between vaccination and testing for BTV3 by grazing as the estimate for Bluevac became higher upon inclusion of grazing in the model, although this numerical difference was not accompanied by a difference in statistical significance of this estimate. Fully grazing herds surprisingly had lower odds of being tested than fully housed herds. This contrasts earlier findings on the BTV8 outbreak in The Netherlands in 2007, where a significantly lower herd seroprevalence was found in herds that kept their cows indoors all summer (25). Another potential confounder is the time between vaccination and exposure (and therefore positive test results). On average, Syvazul vaccinations were given first, followed by Bluevac and then by Bultavo. Possibly, the immunity of Syvazul and to lesser extent Bluevac was weakened in the time to exposure. On the other hand, for every vaccine, some herds already tested positive before the second vaccination or within the period of 21 days after the second vaccination, so prior to full protection. We believe therefore that the time between vaccination and exposure was relatively short and this may only partly explain differences in vaccine efficacy.

It is well described in literature that inactivated bluetongue vaccines against other serotypes are capable of a total prevention of viremia in cattle and sheep (9, 26–28). For the BTV3/NET2023 strain, however, a total prevention of viremia by vaccination seems challenging. None of the three provisionally allowed vaccines claimed that they prevent viremia (29), although for Bultavo an absence of viremia after experimental challenge in calves has been shown (30). Possibly, the strain used in the Bultavo vaccine (Bio-93:BTV3) elicits a more protective immune response than the BTV3/NET2023 strain, used in the other two vaccines. Supportive of our findings, previous work has shown a clear neutralizing antibody response by Bultavo and a more dominant general antibody response by the other two vaccines (31).

### NSAID usage

Non-vaccinated herds had on average 2.59 more treatment days with NSAID per 10 cows in q3 of 2024 compared to the same period a year before. In Bluevac and Syvazul vaccinated herds, this increase was even bigger, but in Bultavo vaccinated herds, a significantly smaller increase was seen. We assume that the increase in NSAID usage is a proxy for an increase in morbidity and that the increase in NSAID usage is likely largely attributable to the BTV3 outbreak as no other reasons for the substantial increase were reported by the practices that provided the data.

However, the fact that NSAID usage was higher for vaccinated herds already in 2023 suggests that farmers who are inclined to vaccinate may also use more NSAID in general, in line with the abovementioned inclination to submit samples for testing. A recent Irish study (34) about dairy farmers’ considerations for NSAID usage in a pasture-based system shows that farmers who give higher pain scores for certain conditions also had higher NSAID use, suggesting that the farmer’s personal view on animal welfare may be linked to use of painkillers. Furthermore, an increased NSAID usage in vaccinating herds may in part have been caused by the fact that at various times both veterinarians and farmers were called upon to report possible reduced efficacy of BTV3 vaccines to the government (32, 33), because these vaccines were approved under exceptional circumstances. These extra contact moments between farmer and veterinarian may have led veterinarians to urge farmers to use NSAID in animals with clinical signs. Such biases complicate the interpretation of our findings. Due to the observational nature of our study, we cannot rule out these and other confounding factors and therefore cannot make absolute claims about the effectiveness of the vaccines to prevent clinical signs. It seems, however, reasonable to assume that the differences between vaccinating farmers and non-vaccinating farmers were not specific to what type of vaccine they used and biases that may explain the higher NSAID usage in 2024 in Bluevac and Syvazul vaccinating herds will also have played a role in Bultavo vaccinating herds. Therefore, the fact that Bluevac and Syvazul were associated with significantly higher difference in NSAID usage between 2024 and 2023 than non-vaccinating herds, whereas Bultavo was associated with significantly lower difference in NSAID usage, suggests that Bultavo gave better protection against clinical disease than the other two vaccines. A waning protection after vaccination as an explanation is a less likely explanation for the a lower performance of Bluevac and Syvazul, since all fully vaccinated herds could not have been vaccinated more than 2 months before the start of 2024 bluetongue outbreak. Another reason for vaccine-specific differences in NSAID usage could be that rumors about limited vaccine efficacy for some of the vaccines used caused farmers and veterinarians to have a closer look at animals vaccinated with these vaccines and therefore use more NSAID as they were more attentive to clinical disease. This may explain some of the overusage in Bluevac and Syvazul vaccinated herds, but it seems unlikely that this fully explains the large difference between Bultavo and the other vaccines.

## Conclusion

Our study suggests substantial differences between vaccines in their ability to prevent positive tests outcomes and clinical signs that warrant NSAID usage within the BTV3/NET2023 outbreak in 2024. Bultavo seemed to perform better at these parameters and is therefore probably preferable to prevent clinical signs and disease transmission.

## Data Availability

The raw data supporting the conclusions of this article will be made available by the authors, without undue reservation.
